# Violence exposure among children in Nigeria

**DOI:** 10.1192/j.eurpsy.2021.1889

**Published:** 2021-08-13

**Authors:** A. Oladosu, O. Abiodun, M. Tunde-Ayinmode

**Affiliations:** 1 Johnson Community Hospital, Lincolnshire Partnership Foundation Trust, Lincolnshire, United Kingdom; 2 Behavioural Sciences, University of Ilorin Teaching Hospital, Ilorin, Nigeria

**Keywords:** violence exposure, home, children

## Abstract

**Introduction:**

There is a paucity of information on the exposure of children to violence in Nigeria. The current study aims, as part of a larger study, to explore the experiece of children to violence in their homes in Nigeria.

**Objectives:**

To determine the prevalence and pattern of violence exposure of children in Ilorin Nigeria.

**Methods:**

Cross sectional survey of 1,554 secondary school students aged 11-18 years in Ilorin Nigeria using multistage random sampling technique with proportional allocation was done. Respondents completed the ICAST-CH questionnaire which covers childrens’ exposue to violence. Prevalence of violence exposure was computed.

**Results:**

63.4% (994/1554) of respondesnts had witnessed violence at home. Table 1: Pattern of violence exposure at home in the last 12 months Form Frequency Percentage*

**Table:** 

Violence Exposure* (n=994)		
Something stolen from home	532	53.5
Adults shouted in a frightening way	392	39.4
Witnessed adults in home hit, kick, slap	378	38.0
Seen people being shot, bombs, rioting	210	21.1
Adults used alcohol then frightened	82	8.2
Someone close got killed near home	56	5.6
Witnessed adults in home use weapons	10	1.0

**Conclusions:**

A good number of children in Nigeria might be exposed to violence. There is thus a need for initiative to strengthen family life and control the exposure of children to violence given its potential to cause long standing mental health problems in victims.

**Disclosure:**

No significant relationships.

